# SNORD76, a box C/D snoRNA, acts as a tumor suppressor in glioblastoma

**DOI:** 10.1038/srep08588

**Published:** 2015-02-26

**Authors:** Luyue Chen, Lei Han, Jianwei Wei, Kailiang Zhang, Zhendong Shi, Ran Duan, Shouwei Li, Xuan Zhou, Peiyu Pu, Jianning Zhang, Chunsheng Kang

**Affiliations:** 1 Laboratory of Neuro-Oncology, Department of Neurosurgery, Tianjin Neurological Institute, Tianjin Medical University General Hospital; 2 Key Laboratory of Neurotrauma, Variation, and Regeneration, Ministry of Education and Tianjin Municipal Government; 3 Chinese Glioma Cooperative Group (CGCG), China; 4 Beijing Tian Tan Hospital, Capital Medical University; 5 Beijing Sanbo Brain Hospital, Capital Medical University

## Abstract

Glioblastoma (GBM) is associated with disproportionately high morbidity and mortality, reflecting the need to develop new diagnostic and therapeutic targets for this disease. Recently, accumulating evidence has suggested that small nucleolar RNAs (snoRNAs) are gaining prominence and are more actively involved in tumorigenesis than previously thought. However, no report concerning the implication of snoRNAs in glioma has been published to date. In our study, SNORD76 was first found to be inversely associated with Hox Transcript Antisense Intergenic RNA (HOTAIR) knockdown, and surprisingly, forcibly expressed SNORD76 inhibited proliferation and growth of glioma cells. Moreover, downregulation of SNORD76 led to a more malignant phenotype. The pleiotropy of SNORD76 overexpression could be achieved at least partially through inducing cell cycle arrest at S phase by affecting the Rb-associated cell cycle regulation. Enforced SNORD76 expression in orthotopic tumors resulted in decreased tumor growth and the reduction of tumor volume. Additionally, in surgically resected glioma tissues, SNORD76, not its host gene, was associated with the WHO classification and was selectively downregulated in GBM (WHO grade IV). Collectively, our study adds to a growing body of evidence for the participation of snoRNAs in gliomagenesis and is the first to implicate a snoRNA in glioblastoma.

Unearthed from the forgotten landscape of ‘dark genomic matter', non-coding RNAs (ncRNAs) have become rising stars in cancer genetics. However, unlike the super star ‘microRNAs', there are relatively few studies on the contributions of small nucleolar RNAs (snoRNAs)^1^,^2^ to the genesis and progression of cancer^3^. snoRNAs are evolutionally conserved small ncRNA molecules of approximately 60–300 nucleotides in length that are predominantly found in the nucleolus and can be divided into two groups according to the rRNA modification type: C/D box snoRNAs (SNORDs) guide 2′-O-methylation while H/ACA box snoRNAs (SNORAs) guide pseudouridylation^4^,^5^,^6^. In the past few years, the common assumption that snoRNAs only act as housekeeping genes has been challenged due to the converging evidence demonstrating that snoRNAs are involved in the control of cell fate and oncogenesis^7^,^8^,^9^,^10^,^11^,^12^,^13^. For example, h5sn2, a H/ACA box snoRNA that is highly expressed in normal brain but is decreased dramatically in meningioma, first linked snoRNA with human malignancy^14^. U50, a potential tumor suppressive C/D box snoRNA identified at locus 6q14.3, was discovered with a preference for a homozygous two-base pair (TT) deletion in prostate cancer^9^, whereas a heterozygous genotype of the deletion occurred more frequently in breast cancer^10^. In addition, SNORD112-114 located at the DLK1-DIO3 locus was found to be overexpressed in acute promyelocytic leukemia (APL) with the potential to affect Rb/p16 cell cycle regulation^12^. These independent reports on snoRNAs provide evidence for the functional importance of snoRNAs in cancer and suggest that, far beyond our imagination, snoRNAs are more actively involved in tumorigenesis.

Glioblastoma multiforme (GBM), the most common and biologically aggressive malignant glioma with a median survival ranging from 12 to 15 months^15^,^16^,^17^, is associated with a deterioration in neurocognitive function, decreased functional independence, and a progressive decrease in health-related quality of life^18^,^19^. Thus far, no further improvements in GBM outcome have been documented since the introduction of radiotherapy-temozolomide therapy in 2005^18^. Therefore, the primary rationales for the precise diagnostic and prognostic biomarkers and effective therapeutic targets for GBMs have been intensely studied by neuro-oncologists worldwide. However, excluding the progress made regarding protein coding genes and miRNAs, little attention has been paid to the oncogenic or tumor suppressive roles of snoRNAs in GBMs. Given the increasing evidence of certain snoRNAs as potential regulators of cell fate and insight into the molecular mechanisms by which snoRNAs may carry out these regulatory functions, research is required to comprehensively understand the mechanisms by which aberrant snoRNAs contribute to the development and progression of cancer.

In the present study, we identified a C/D box snoRNA U76 (SNORD76), a small nucleolar RNAs excised from the third intron of growth arrest-specific transcript 5 (GAS5), while performing transcriptome sequencing for differently expressed genes after Hox Transcript Antisense Intergenic RNA (HOTAIR) knockdown (data not shown). Our previous work on HOTAIR identified HOTAIR is an independent prognostic factor and a cell cycle-associated long ncRNA^20^. Thus, we hypothesized that SNORD76, whose expression was elevated after HOTAIR knockdown, might act as a tumor suppressive gene that is suppressed by HOTAIR-mediated epigenetic modification, or an oncogene that compensates for the loss of HOTAIR, rather than a housekeeping gene in GBM. Surprisingly, we found that forced SNORD76 expression inhibits the tumorigenicity of glioma cells by inducing retinoblastoma gene (Rb)-associated cell cycle arrest. Additionally, in surgically resected brain tumor specimens, SNORD76 expression was significantly decreased in GBM (WHO grade IV). Therefore, SNORD76 may have a tumor suppressive role in brain tumorigenesis by arresting tumor cells in S phase, rather than just a simple reflection of the cellular stress or a secondary effect of cancer transformation.

## Results

### SNORD76 expression inversely associates with HOTAIR alteration in glioma cells

**Figure 1:**
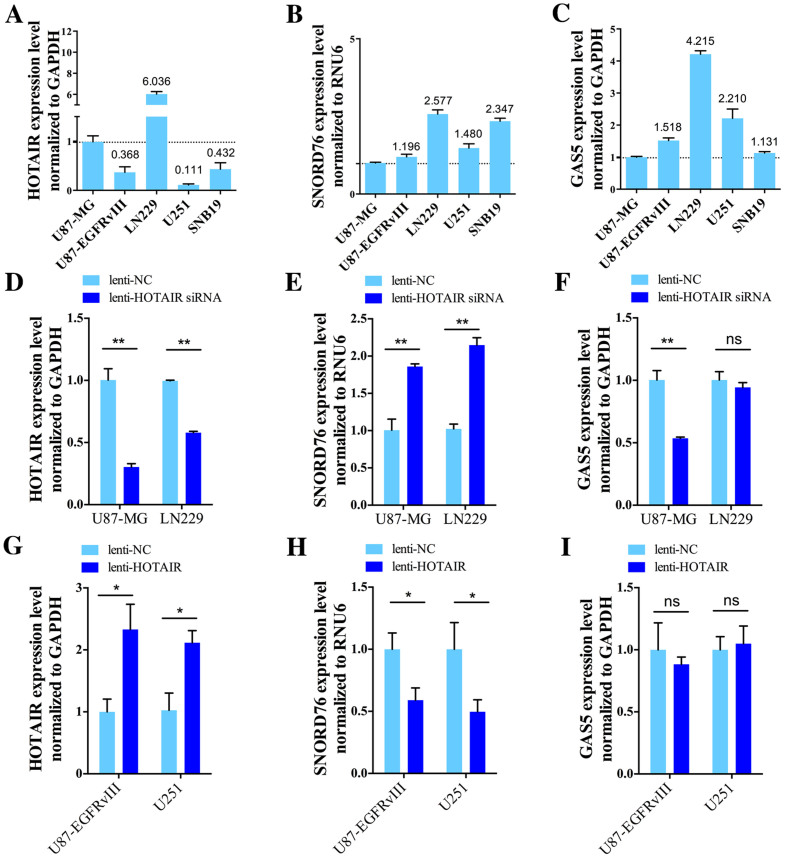
SNORD76, rather than its host gene (GAS5), is elevated after HOTAIR knockdown in glioma cell lines. Detection of HOTAIR (A), SNORD76 (B) and GAS5 (C) expression by qRT-PCR in glioma cell lines. Numbers above solid bars are the relative expression when compared to U87-MG. (D) Quantitative PCR validation of HOTAIR knockdown efficacy in LN229 and U87-MG glioma cell lines. (E) and (F) Expression of SNORD76 and GAS5 as determined by qPCR after HOTAIR siRNA treatment. The expression of HOTAIR (G), SNORD76 (H), and GAS5 (I) was measured by qPCR after infection with lentivirus expressing the full length of HOTAIR. All data represent means ± s.e.m. of three experiments performed in triplicate. *P < 0.05, **P < 0.01 (Student's *t* test), ns, no significant difference (*P ≥ 0.05*) relative to the control group.

To determine whether the increase of SNORD76 following the HOTAIR siRNA-expressing lentivirus treatment was coincidental, we used quantitative PCR to evaluate the expression of HOTAIR, SNORD76, and GAS5 in 5 glioma cell lines (U87, U87-EGFRvIII, LN229, U251 and SNB19). In the 5 examined glioma cell lines, LN229 and U87-MG expressed higher HOTAIR, while lower SNORD76 expression was found in U87-MG and U87-EGFRvIII (Fig. 1A–C). However, U87-EGFRvIII, a cell line derived from U87-MG by stably expressing EGFRvIII mutant, may have the similar cellular background with U87-MG. Thus, in the present study, we decided to perform HOTAIR knockdown experiments in LN229 and U87-MG, and then SNORD76 overexpression in U87-MG and U251. In the same manner, HOTAIR overexpression experiments were performed in U251 and U87-EGFRvIII, and SNORD76 knockdown in LN229 and SNB19.

After infection with a lentivirus expressing HOTAIR siRNA, the HOTAIR expression reduced by approximately 50% in U87-MG and LN229 (Fig. 1D, *p* < 0.01). Consequently, SNORD76 in both cell lines increased by approximately 2-fold (Fig. 1E, *p* < 0.01); however, in U87-MG, GAS5 showed an unexpected decrease, while in LN229 no significant change was detected (Fig. 1F). On the contrary, forced HOTAIR expression by lentiviruses expressing full transcript of HOTAIR led to a significant decrease in SNORD76 expression while no significant change in GAS5 expression was observed (Fig. 1G, *p* < 0.05; 1H, *p* < 0.05; 1I, *p* > 0.05). Therefore, we deduced that HOTAIR regulates the expression of SNORD76 through a previously undetermined mechanism rather than the canonical role in mediating epigenetic modification (see the discussion). In addition, disproportional expressions of GAS5 and its embedded snoRNAs after interventions have been previously reported by other studies^8^,^21^.

### SNORD76 inhibits cell growth and proliferation *in vitro*

**Figure 2:**
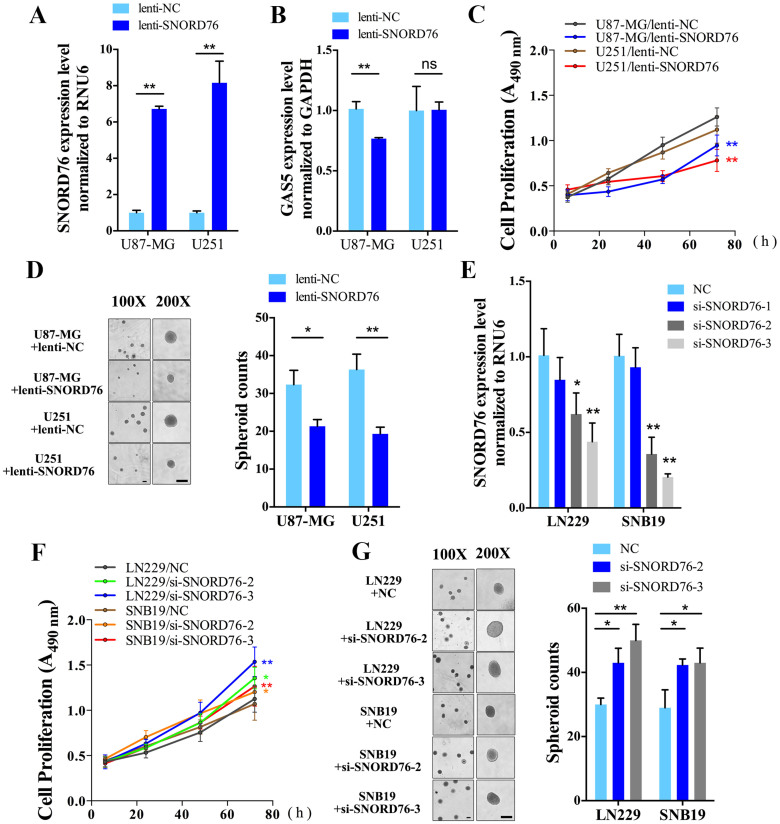
SNORD76 inhibits cell growth and proliferation of glioma cell lines. (A) Determination of SNORD76 expression by qPCR and the corresponding expression of GAS5 (B) after 48 h infection with lentivirus encoding the full-length transcript of SNORD76 (lenti-SNORD76). (C) Proliferation rates of glioma cells infected with a SNORD76-expressing lentivirus were measured by MTT assay (n = 6). (D) Anchorage-dependent growth of glioma cells was determined by soft-agar colony formation assay (n = 3). The left panel was representative image taken by phase contrast microscope, and the right panel was total spheroid count in 5 randomly chosen fields at 100× magnification. (E) Knocking-down efficacy of three different SNORD76 siRNA sequences. Cell proliferation rates (F) and anchorage-dependent growth (G) were evaluated after SNORD76 siRNA treatment. Error bars represent s.d (C, D, F, G) and s.e.m (A, B, E). The black scale bar corresponds to 50 μm. *P < 0.05, **P < 0.01 (Student's *t* test), ns, no significant difference (*P ≥ 0.05*) relative to the control group.

As increasing evidence suggests that snoRNAs are more than monotonous elements in post-transcriptional modification and have been implicated in cancer development and progression, we therefore constructed a lentivirus vector encoding a full-length transcript of SNORD76 to verify whether SNORD76 plays a potentially functional role in glioma. After a 48 h infection, SNORD76 expression levels in both U87-MG and U251 increased by over 6-fold (Fig. 2A, *p* < 0.01). Interestingly, only a slight change in GAS5 expression was observed in both cell lines (Fig. 2B).

To study the biological implication of SNORD76, we performed MTT and soft-agar colony formation assay to determine SNORD76's influence on glioma cell proliferation and growth. Forced SNORD76 expression in U87-MG and U251 reduced the proliferation rate of both cell lines (Fig. 2C). In addition, anchorage-independent growth was also impaired in SNORD76 overexpressing groups, which were represented by a lower volume and number of spheroids compared with cancer cells infected with a nonsense control (Fig. 2D, *p* < 0.05). Prior to performing SNORD76 loss-of-function experiments, we examined the knockdown efficacy of the three designed siRNAs targeting SNORD76. Both the si-SNORD76-2 and si-SNORD76-3, which could significantly decrease the expression of SNORD76 in both cell lines, were chosen to knockdown SNORD76 in the follow experiments to evade the potential off-target effect(Fig. 2E, *p* < 0.05). Subsequently, in LN229 and SNB19 that express higher endogenous SNORD76, knockdown of SNORD76 using the selected siRNAs increased the proliferation rate and anchorage-independent growth, which led to a more malignant phenotype (Fig. 2F–G). The observations from the gain- and loss-of-function experiments were previously unknown, and hence, the possible underlying mechanism needs to be explored.

### Ectopic expression of SNORD76 inhibits tumorigenicity by arresting cancer cells in S phase of the cell cycle

**Figure 3:**
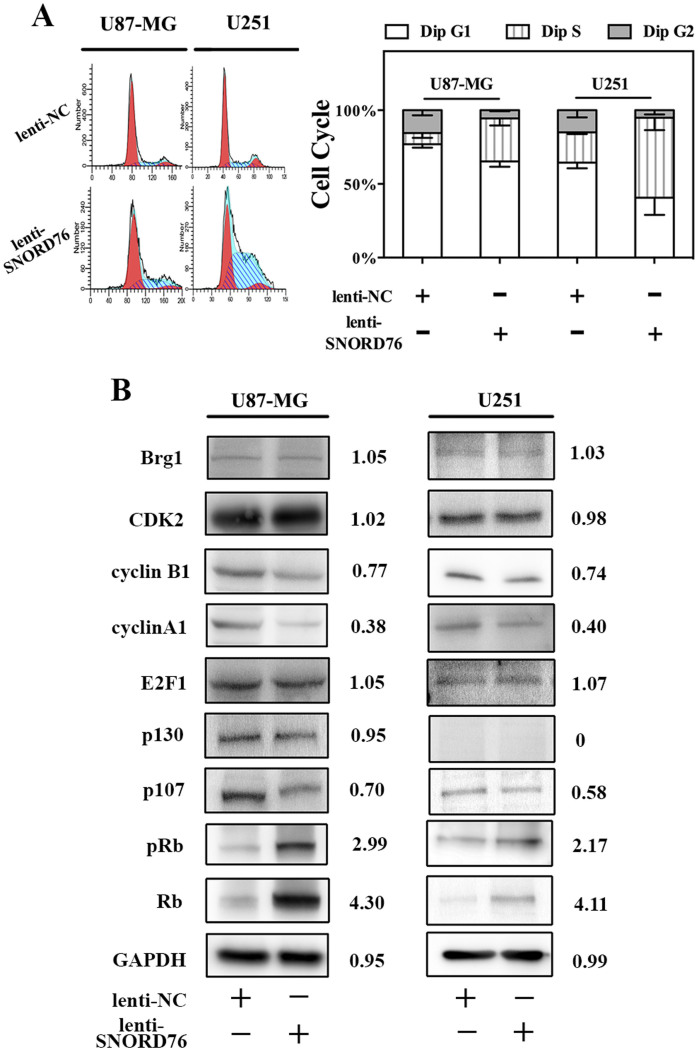
Overexpression of SNORD76 arrests glioma cells at S phase. (A) Cell cycle distribution analysis of U87-MG and U251 glioma cells infected with lenti-NC or lenti-SNORD76 (n = 3). (B) Western blot analysis of the cell cycle associated proteins.

The above observation demonstrated that enforced SNORD76 expression inhibited the growth and proliferation of glioma cells. However, the underlying mechanism is still unknown. Propidium iodide staining with flow cytometry analysis showed that overexpressing SNORD76 resulted in the accumulation of cells in S phase at 48 h following lentivirus infection. Additionally, consistent with a previously characterized tumor suppressive role in glioma, exit from S phase was seemingly blocked after SNORD76 treatment because there was no significant increase in the G2 phase (Fig. 3A). It is clear that controlling the progression of cells into and through the S phase of the cell cycle is important in regulating DNA synthesis and thus cell proliferation. Fluctuations of cyclin A1 and cyclin B1 normally reach a peak in the G2-M transition of the cell cycle^22^, and in this study, the expression levels of the cyclins were downregulated significantly in the glioma cells overexpressing SNORD76 (Fig. 3B), corresponding to a cyclin-expression pattern of S phase arrested cells. Intriguingly, Rb and p107 of the Rb family, rather than p130, were expressed differently in SNORD76-treated cells, with an increased expression of Rb and decreased p107 (Fig. 3B). p130 expression could not be detected in the U251 cell line. Additionally, the hyperphosphorylated Rb (pRb) expression was paradoxically elevated in SNORD76-overexpressing cells. Other cell cycle related proteins, such as CDK2, Brg1 and E2F1, remained unchanged. Our results are consistent with a previous study by Zhang *et al*^23^, which showed that Rb can form a complex with Brg1 that cooperatively inhibits exit from S phase. Therefore, our observations demonstrated that SNORD76 overexpression inhibits the tumorigenicity of glioma cells by affecting the Rb-associated cell cycle regulation.

### Forced SNORD76 expression in U87-MG cells impedes orthotopic tumor growth *in vivo*

**Figure 4:**
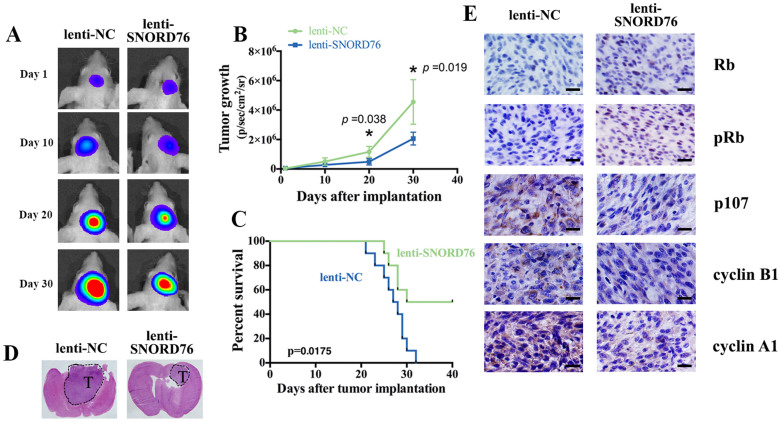
SNORD76 overexpression impairs *in vivo* tumorigenicity of glioma cells. (A) Representative images of bioluminescence in mice on days 1, 10, 20, and 30 after implantation. (B) Quantitative analysis of these images revealed inhibited growth of the lenti-SNORD76 treated U87-MG tumors *in vivo* (n = 3). (C) Animal survival analysis (n = 10, Log-rank (Mantel-Cox) test). (D) Hematoxylin and eosin staining (H&E) of the tumor (marked with ‘T') derived from U87-MG cells. (E) Immunohistochemical staining (IHC) of the proteins that were previously found to be altered in response to SNORD76 overexpression *in vitro*. The black scale bar corresponds to 200 μm. All data represent means ± s.d. *P < 0.05 (Student's *t* test).

Given that SNORD76 inhibited tumorigenicity *in vitro*, we hypothesized that SNORD76 would be associated with a repression of *in vivo* tumorigenicity. Prior to implantation, we co-infected U87-MG tumor cells with lentiviruses expressing luciferase and a nonsense control or SNORD76 for 48 h, and then intracranially injected the pre-treated cells into nude mice. *In vivo* imaging analysis of the mice 1, 10, 20 and 30 days after implantation revealed that the growth of orthotopic tumors was significantly inhibited by forced expression of SNORD76 (Fig. 4A–B, *p* < 0.05). Likewise, treating with SNORD76 was associated with a significantly longer survival rate of the mice (Fig. 4C, *p* < 0.05). Similarly, hematoxylin and eosin staining of the brain tissues resected from the orthotopic models showed that the SNORD76-treated tumor volume was reduced when compared to those treated with nonsense control (Fig. 4D). Furthermore, IHC showed decreased expression of cyclin A1, cyclin B1 and p107 and the increased expression of Rb and pRb, which were consistent with the *in vitro* results (Fig. 4E). Altogether, these *in vivo* findings demonstrate that SNORD76 overexpression inhibits *in vivo* tumorigenicity of glioma cells.

### SNORD76 downregulation is associated with an aggressive phenotype in glioma

**Figure 5:**
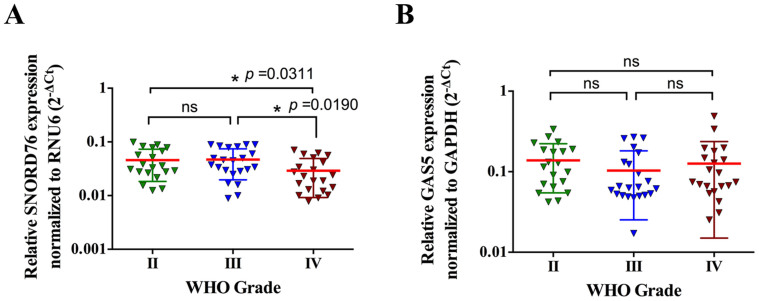
SNORD76 expression, rather than its host gene (GAS5), is downregulated in surgically resected glioblastoma (WHO grade IV) tissues. (A) Compared with glioma tissues of WHO grade II (n = 20) and grade III (n = 22), tissues from glioblastoma (n = 21) displayed lower SNORD76 expression as determined by qPCR. (B) No clinically significant difference in GAS5 expression among tested glioma tissues of WHO grade II, III and IV was detected. *P < 0.05 (ANOVA); ns, no significant difference (*P ≥ 0.05*) relative to the other group.

To investigate the clinical relevance of SNORD76 in gliomas, we performed quantitative PCR on surgically resected glioma tissues of WHO grade II (n = 20), grade III (n = 22), and grade IV (n = 21). Overall, compared with gliomas of WHO grade II and III, SNORD76 displayed a significantly lower expression in glioblastoma (WHO grade IV), while there was no significant difference between tumors with lower grades (Fig. 5A, *p* < 0.05). In addition, the expression of GAS5, the host gene of SNORD76, showed no significant differences in the clinical samples (Fig. 5B). Collectively, detection of SNORD76 expression might potentially be a usable biomarker for glioblastoma.

## Discussion

In the past two decades, there has been important progress in our understanding of the molecular pathogenesis of malignant gliomas, whose malignant transformation results from the sequential accumulation of genetic aberrations and the dysregulation of growth-factor signaling pathways^17^. However, little light has been shed on the non-protein coding genome or transcriptome in cancer, except for miRNAs, which are well characterized by target recognition and regulatory functions^24^,^25^. It is not until recently that snoRNAs have been justified to have a functional role in carcinogenesis. Despite the emerging insights into the intriguing new roles for snoRNAs in various types of cancer, there are no previous reports about snoRNAs in gliomas. In our study, we found that interventions on the expression of HOTAIR placed an adverse impact on SNORD76. However, this regulatory effect might be executed beyond the canonical function as a scaffold molecule for the histone methyltransferase complex, transcriptionally repressing the HOTAIR-targeting genes^26^. SNORD76, located in the third intron of the GAS5 DNA sequence^27^, is a “by-product” of appropriately processing nascent GAS5 RNA (pre-RNA) into mature GAS5 RNA, which means that the alterations in the expression of GAS5 and SNORD76 will be consistent once they were regulated by HOTAIR transcriptionally. Based on our observations, GAS5 was barely affected by the altered expression of HOTAIR while SNORD76 was negatively associated with HOTAIR. LncRNAs, such as MALAT-1, have been previously reported to regulate alternative splicing of pre-RNA that will lead to an increasing amount of dissociated segments that encoding by the introns, of which can be further processed into mature snoRNAs^28^. Therefore, we deduced that HOTAIR possibly regulates SNORD76 in a non-canonical mechanism – splicing regulation other than epigenetic silencing. This phenomenon can be supported by previous reports of disproportionate expression patterns between snoRNAs and their host genes after the same intervention^21^,^29^.

SNORD76 has no known function in cancer other than the canonical function of rRNA 2′-O-methylation, whereas HOTAIR, a long intervening non-coding RNA mediating epigenetic modification, was previously reported to be associated with poor prognosis and histological grade in gliomas^20^. We performed gain- and loss-of-function experiments to explore the possible functional role of SNORD76 in glioma tumorigenesis. Overexpression of SNORD76 by a lentivirus vector could suppress the proliferation and growth of low endogenous SNORD76 glioma cell lines and, in contrast, suppression of SNORD76 in higher SNORD76 cell lines led to a more malignant phenotype. Collectively, we suggested that this small nucleolar RNA, SNORD76, could be a tumor suppressor in glioma rather than a monotonous element in response to oncogenic HOTAIR alterations. Even though the anti-tumor effect of SNORD76 was observed in glioma *in vitro*, there is still, so far, no clear explanation for this phenomenon. PI-staining flow cytometry analysis showed that forced SNORD76 expression arrested glioma cells at S phase of the cell cycle, providing a clue that SNORD76 may affect the expression of cell cycle-associated proteins. It has been documented that suppression of oncogenic SNORD42 in non-small cell lung cancer could increase apoptosis through a p53-dependent manner^8^ and SNORD114-1 promotes the cell growth through affecting the Rb/p16 cell cycle regulation^12^. Western blot analysis of cell cycle-associated proteins, especially focused on those related to the regulation of the S/G2 phase of the cell cycle, found that the expressions of Rb, pRb, p107, cyclin A1 and cyclin B1 were altered after SNORD76 overexpression. The expression of Rb and pRb increased by ectopic SNORD76 expression, which in turn, led to cell cycle arrest at S phase. Phosphorylation of Rb protein releases the G1 blockade that in turn, increases the G1/S phase transition. Moreover, Rb has been reported to act as a key regulator of the exit from G1 and S phase of cell cycle by forming Rb-E2F-HDAC (histone deacetylase) and Rb-Brg1 repressive complex, respectively^23^,^30^. In lines with S phase arrest, the expression of cyclin A1 and cyclin B1, which peaked at G2-M phase of the cell cycle, was significantly reduced after SNORD76 overexpression^31^. However, the mechanism by which SNORD76 increased the expression of Rb remained unknown. Recently, a paper published on *Cell* extended pseudouridylated types of RNA to mRNA, which was previously characterized as a typical modification in ribosomal RNA and spliceosomal RNA. Pseudouridylation of mRNA by the H/ACA box snoRNAs (SNORAs) enhances the transcript stability^32^. Being another essential component of snoRNAs, we therefore deduced that some of the C/D box snoRNAs (SNORDs), the key mediators of rRNA 2′-O-methylation, might also play a previously unrecognized role in mRNA modification, and thus increase the mRNA stability.

It is attractive to further identify whether enforced SNORD76 expression could impede tumor growth *in vivo*, because SNORD76 was shown to inhibit tumor growth and proliferation *in vitro*. Surprisingly, in orthotopic mouse models, the growth of tumors derived from SNORD76 overexpressing U87-MG cells was significantly inhibited and importantly, SNORD76-treated mice demonstrated a more favorable outcome when compared to the mice treated with the nonsense control. We supposed that impairments of cell growth and proliferation that alleviate the neoplastic extrusion towards normal brain were responsible for this difference in survival. Both the *in vitro* and *in vivo* suppression of glioma by overexpressing SNORD76 laid the groundwork for SNORD76 to be developed into a promising therapeutic or ancillary target for effectively combined treatment in the future. In addition, despite the works on glioma cell lines, SNORD76 was found to express exclusively lower in glioblastoma samples (WHO grade IV) than those of the other histological grades (WHO grade II and III), which gave rise to the possibility that SNORD76 could be used as a specific biomarker for GBM.

In summary, HOTAIR could regulate the expression of SNORD76 independent of regulation of the expression of GAS5, and SNORD76 caused marked repression of glioma growth *in vitro* and *in vivo*. The tumor suppression could be achieved at least partially through inducing S phase arrest in a Rb-associated manner. SNORD76 may act as a potential tumor suppressive gene in the development and progression of glioblastoma and may be a potential specific glioblastoma biomarker. Though our findings first link snoRNAs with the gliomagenesis, we have to recognize that more works need to be done to identify the underlying mechanisms.

## Methods

### Cell culture

Human U87-MG, LN229, U251, and SNB19 GBM cells were obtained from the China Academia Sinica Cell Repository (Shanghai, China). U87-EGFRvIII was a kind gift from Prof. Ren Huan (Harbin Medical University). The GBM cells were maintained in Dulbecco's modified Eagles medium (DMEM; Gibco) supplemented with 10% fetal bovine serum (FBS; Gibco), and additionally 400 μg/mL G418 (EMD bioscience) for U87-EGFRvIII at 37°C in a 5% CO_2_ atmosphere in a humidified chamber.

### Clinical specimens

Freshly resected tissue was immediately frozen in liquid nitrogen for subsequent total RNA extraction. All grades of glioma specimens with clinical data were collected from Beijing Sanbo Brain Hospital. The study was approved by the hospital institutional review board, and written informed consent was obtained from all of the patients.

### RNA extraction and quantitative real-time PCR analysis

Total RNA was extracted using TRIzol (Invitrogen). qRT-PCR assays were performed to measure the expression levels of SNORD76, HOTAIR, and GAS5. Real-time PCR was performed using the SYBR Green PCR Master Mix (Applied Biosystems) according to the manufacturer's instructions. Primers specific for each of the signaling molecules were designed using NCBI/Primer-BLAST and used to generate the PCR products. Expression levels of GAPDH were used for normalization and quantification of HOTAIR and GAS5 expression levels, while the expression level of RNU6 was used for SNORD76 expression quantification. For the quantification of gene amplification, real-time PCR was performed using the DNA Engine Opticon 2 Two-Color Real-Time PCR detection system (Bio-Rad Laboratories) in the presence of SYBR-Green. Real-time PCR data were analyzed by the comparative Ct method^33^. The following gene-specific primers were used: SNORD76 (F: 5′-TGACAGTTTATTTGCTACTCTTGAG-3′; R: 5′- TAAGATAATGGTGGTTAAGATCCTC-3′); GAS5 (F: 5′-CTTCTGGGCTCAAGTGATCCT-3′; R: 5′-TTGTGCCATGAGACTCCATCAG-3′); HOTAIR (F: 5′-GAGAAAAGGCTGAAATGGAGGACC-3′; R: 5′-TCTTCCCTCCTCTGGCTCTCTCTC-3′); GAPDH (F: 5′-CTCAAGGGCATCCTGGGCTAC-3′; R: 5′-CAGCCCCAGCGTCAAAGGT-3′); RNU6 (F: 5′-ATTGGAACGATACAGAGAAGATT-3′; R: 5′-GGAACGCTTCACGAATTTG-3′).

### Lentivirus preparation and *in vitro* infection

Lentiviral vectors expressing nonsense control (NC), HOTAIR siRNA (sense: 5′-CCACAUGAACGCCCAGAGA-3′), SNORD76 (NCBI Reference Sequence: NR_003942.1), HOTAIR (NCBI Reference Sequence: NR_047518.1) or luciferase were all generated by GenePharma (Shanghai, China). Cell infections were carried out according to GenePharma's recommendations.

### RNA interference

Three pairs of siRNAs designed to knockdown SNORD76 expression and one pair of non-sense control RNA were purchased from Ribobio (Guangzhou, China). Transfection was performed by using Opti-MEM medium and Lipofectamine 3000 (Invitrogen) according to the manufacturer's protocol. siRNA targeted sequences are listed below: si-SNORD76-1 (5′-CCACAATGATGACAGTTTA-3′), si-SNORD76-2 (5′-GCTACTCTTGAGTGCTAGA-3′), and si-SNORD76-3 (5′-GAGGATCTTAACCACCATT-3′).

### Cell cycle analysis

Glioma cells were infected with lenti-SNORD76 or lenti-nonsense control. After fixation in 70% ethanol and RNase A treatment, cells were stained with propidium iodide. DNA content was analyzed by flow cytometry.

### MTT assay

Cell growth rates were measured by MTT assay. Cells were plated in 96-well plates and treated as indicated. Following treatment, each well was incubated with 20 μL of 5 mg/mL 3-[4,5-dimethylthiazol-2yl]-2,5-diphenyl-tetrazolium bromide (MTT) for 4 h in a CO_2_ incubator at 37°C. The medium was aspirated and 0.2 mL DMSO was added per well. Proliferation rates were measured by a colorimetric assay of formazan intensity in a plate reader at 490 nm.

### Soft-agar colony formation assay

Anchorage-independent growth was evaluated by the clonogenicity of cells on soft-agar. Briefly, plates were precoated with 0.7% agarose as the bottom layer. Cells were seeded at a density of 1000 cells per 24-well in triplicate for each cell line and cultured in 0.35% agarose as the top layer in DMEM plus 10% FBS at 37°C for 10 days. The cells were kept wet by adding a small amount of culture media. Colony numbers were counted under the microscope at 100× magnification.

### Western blot analysis and immunohistochemistry (IHC)

Western blotting and IHC assays were performed as previously described^34^. The antibodies used were anti-Rb, anti-pRb, anti-E2F1, and anti-GAPDH from Cell Signaling Technology; anti-CDK2, anti-cyclin A1, and anti-cyclin B1 from Santa Cruz Biotechnology; anti-p107 and anti-p130 from Abcam; and anti-Brg1 from Bioss. Then, expression of GAPDH was used as an internal control.

### Nude mouse tumor intracranial model

The orthotopic tumor models were constructed as previously described^35^. Paraffin-embedded sections (8 μm) were stained with hematoxylin and eosin (H&E) and used for IHC analysis. When experimental animals were used, the research followed the internationally recognized guidelines on animal welfare and local and national regulations. The overall survival curves were plotted according to the Kaplan-Meier method.

### Statistical analysis

Statistical analysis was performed using the SPSS Graduate Pack, version 11.0, statistical software (SPSS). Data are presented as means ± s.e.m. of three independent experiments or means ± s.d. performed in triplicate. One-way ANOVA was used for comparison among the different groups. When the ANOVA was significant, *post hoc* testing of differences between groups was performed using a LSD test. A P-value < 0.05 was considered to be significant.

## References

[b1] Lestrade L. & Weber M. J. snoRNA-LBME-db, a comprehensive database of human H/ACA and C/D box snoRNAs. Nucleic Acids Res 34, D158–62 (2006). 16381836 10.1093/nar/gkj002 PMC1347365

[b2] Makarova J. A. & Kramerov D. A. SNOntology: Myriads of novel snoRNAs or just a mirage? BMC Genomics 12, 543 (2011). 22047601 10.1186/1471-2164-12-543 PMC3349704

[b3] Williams G. T. & Farzaneh F. Are snoRNAs and snoRNA host genes new players in cancer? Nat. Rev. Cancer 12, 84–88 (2012). 22257949 10.1038/nrc3195

[b4] Kiss T. Small nucleolar RNAs: an abundant group of noncoding RNAs with diverse cellular functions. Cell 109, 145–148 (2002). 12007400 10.1016/s0092-8674(02)00718-3

[b5] Henras A. K., Dez C. & Henry Y. RNA structure and function in C/D and H/ACA s(no)RNPs. Curr. Opin. Struct. Biol. 14, 335–343 (2004). 15193314 10.1016/j.sbi.2004.05.006

[b6] Kiss T. Small nucleolar RNA-guided post-transcriptional modification of cellular RNAs. EMBO J 20, 3617–3622 (2001). 11447102 10.1093/emboj/20.14.3617 PMC125535

[b7] Liao J. *et al.* Small nucleolar RNA signatures as biomarkers for non-small-cell lung cancer. Mol. Cancer 9, 198 (2010). 20663213 10.1186/1476-4598-9-198 PMC2919450

[b8] Mei Y.-P. *et al.* Small nucleolar RNA 42 acts as an oncogene in lung tumorigenesis. Oncogene 31, 2794–2804 (2012). 21986946 10.1038/onc.2011.449 PMC4966663

[b9] Dong X. Y. *et al.* SnoRNA U50 is a candidate tumor-suppressor gene at 6q14.3 with a mutation associated with clinically significant prostate cancer. Hum. Mol. Genet. 17, 1031–1042 (2007). 10.1093/hmg/ddm375 PMC2923223 18202102

[b10] Dong X.-Y. *et al.* Implication of snoRNA U50 in human breast cancer. J Genet Genomics 36, 447–454 (2009). 19683667 10.1016/S1673-8527(08)60134-4 PMC2854654

[b11] Su H. *et al.* Elevated snoRNA biogenesis is essential in breast cancer. Oncogene 33, 1348–1358 (2014). 23542174 10.1038/onc.2013.89

[b12] Valleron W. *et al.* Specific small nucleolar RNA expression profiles in acute leukemia. Leukemia 26, 2052–2060 (2012). 22522792 10.1038/leu.2012.111

[b13] Liuksiala T. *et al.* Overexpression of SNORD114-3 marks acute promyelocytic leukemia. Leukemia 28, 233–236 (2014). 23979522 10.1038/leu.2013.250

[b14] Chang L.-S., Lin S.-Y., Lieu A.-S. & Wu T.-L. Differential expression of human 5S snoRNA genes. Biochem Biophys Res Commun 299, 196–200 (2002). 12437969 10.1016/s0006-291x(02)02623-2

[b15] Furnari F. B. *et al.* Malignant astrocytic glioma: genetics, biology, and paths to treatment. Genes Dev 21, 2683–2710 (2007). 17974913 10.1101/gad.1596707

[b16] Bondy M. L. *et al.* Brain tumor epidemiology: consensus from the Brain Tumor Epidemiology Consortium. Cancer 113, 1953–1968 (2008). 18798534 10.1002/cncr.23741 PMC2861559

[b17] Meyer M. A. Malignant gliomas in adults. N. Engl. J. Med. 359, 1850–1850 (2008). 10.1056/NEJMc086380 18946076

[b18] Chinot O. L. *et al.* Bevacizumab plus radiotherapy-temozolomide for newly diagnosed glioblastoma. N. Engl. J. Med. 370, 709–722 (2014). 24552318 10.1056/NEJMoa1308345

[b19] Giovagnoli A. R., Silvani A., Colombo E. & Boiardi A. Facets and determinants of quality of life in patients with recurrent high grade glioma. J. Neurol. Neurosurg. Psychiatr. 76, 562–568 (2005). 15774446 10.1136/jnnp.2004.036186 PMC1739577

[b20] Zhang J.-X. *et al.* HOTAIR, a cell cycle-associated long noncoding RNA and a strong predictor of survival, is preferentially expressed in classical and mesenchymal glioma. Neuro Oncol 15, 1595–1603 (2013). 24203894 10.1093/neuonc/not131 PMC3829598

[b21] Zhang Z. *et al.* Negative regulation of lncRNA GAS5 by miR-21. Cell Death Differ. 20, 1558–1568 (2013). 23933812 10.1038/cdd.2013.110 PMC3792431

[b22] Giacinti C. & Giordano A. RB and cell cycle progression. Oncogene 25, 5220–5227 (2006). 16936740 10.1038/sj.onc.1209615

[b23] Zhang H. S. *et al.* Exit from G1 and S phase of the cell cycle is regulated by repressor complexes containing HDAC-Rb-hSWI/SNF and Rb-hSWI/SNF. Cell 101, 79–89 (2000). 10778858 10.1016/S0092-8674(00)80625-X

[b24] Pasquinelli A. E. MicroRNAs and their targets: recognition, regulation and an emerging reciprocal relationship. Nat Rev Genet 13, 271–282 (2012). 22411466 10.1038/nrg3162

[b25] Bartel D. P. MicroRNAs: target recognition and regulatory functions. Cell 136, 215–233 (2009). 19167326 10.1016/j.cell.2009.01.002 PMC3794896

[b26] Wapinski O. & Chang H. Y. Long noncoding RNAs and human disease. Trends Cell Biol. 21, 354–361 (2011). 21550244 10.1016/j.tcb.2011.04.001

[b27] Yoshihama M., Nakao A. & Kenmochi N. snOPY: a small nucleolar RNA orthological gene database. BMC Res Notes 6, 426 (2013). 24148649 10.1186/1756-0500-6-426 PMC4015994

[b28] Wang K. C. & Chang H. Y. Molecular Mechanisms of Long Noncoding RNAs. Mol. Cell 43, 904–914 (2011). 21925379 10.1016/j.molcel.2011.08.018 PMC3199020

[b29] Askarian-Amiri M. E. *et al.* SNORD-host RNA Zfas1 is a regulator of mammary development and a potential marker for breast cancer. RNA 17, 878–891 (2011). 21460236 10.1261/rna.2528811 PMC3078737

[b30] Kastan M. B. & Bartek J. Cell-cycle checkpoints and cancer. Nature 432, 316–323 (2004). 15549093 10.1038/nature03097

[b31] Sherr C. J. Cancer cell cycles. Science 274, 1672–1677 (1996). 8939849 10.1126/science.274.5293.1672

[b32] Schwartz S. *et al.* Transcriptome-wide mapping reveals widespread dynamic-regulated pseudouridylation of ncRNA and mRNA. Cell 159, 148–162 (2014). 25219674 10.1016/j.cell.2014.08.028 PMC4180118

[b33] Schmittgen T. D. & Livak K. J. Analyzing real-time PCR data by the comparative CT method. Nat Protoc 3, 1101–1108 (2008). 18546601 10.1038/nprot.2008.73

[b34] Zhou X. *et al.* Downregulation of miR-21 inhibits EGFR pathway and suppresses the growth of human glioblastoma cells independent of PTEN status. Lab. Invest. 90, 144–155 (2010). 20048743 10.1038/labinvest.2009.126

[b35] Chen L. *et al.* A lentivirus-mediated miR-23b sponge diminishes the malignant phenotype of glioma cells in vitro and in vivo. Oncol Rep 31, 1573–1580 (2014). 24503899 10.3892/or.2014.3012

